# A systematic review and meta-analysis of topoisomerase inhibition in pre-clinical glioma models

**DOI:** 10.18632/oncotarget.24334

**Published:** 2018-01-29

**Authors:** Toni Rose Jue, Emily S. Sena, Malcolm R. Macleod, Kerrie L. McDonald, Theodore C. Hirst

**Affiliations:** ^1^ Cure Brain Cancer Neuro-Oncology Group, Prince of Wales Clinical School, UNSW Sydney, Australia; ^2^ Center for Clinical Brain Sciences, University of Edinburgh, Edinburgh, UK; ^3^ Department of Neurosurgery, Royal Victoria Hospital, Belfast, UK

**Keywords:** topoisomerase, glioma, systematic review, meta-analysis, meta-regression

## Abstract

Malignant glioma is a devastating disease affecting both adults and children with limited treatment strategies. Pre-clinical animal studies are critical to the development and planning of novel treatment designs for human clinical trials. Topoisomerases has been a target of interest in the treatment of high grade gliomas, such as glioblastoma, in the past years. Here we assess pre-clinical glioma literature with the aim to identify predictive variables that favour treatment outcomes from topoisomerase inhibition. Data was extracted from 90 experimental comparisons, this was divided based on available survival (*n* = 61) and tumor volume (*n* = 29) data. The meta-analysis revealed that the overall effect of topoisomerase inhibition prolonged survival by a factor of 1.33 (95% CI: 1.23–1.43) and reduced tumor growth by a factor of 3.21 (95% CI: 1.99–5.88), with considerable between-study heterogeneity. Multivariable meta-regression identified glioma model, type of control, route of drug administration and drug of choice to be predictive of improved survival outcome. Publication bias assessment by contour-enhanced funnel plots, Egger’s regression test and trim and fill analysis showed evidence of publication bias in all studies. This study identified multiple study design factors that should be taken into consideration to improve the translation of pre-clinical investigation of topoisomerase inhibition into clinical use.

## INTRODUCTION

Glioblastoma (GBM) is the most common malignant brain tumor with an incidence rate of 3.20 per 100,000 population [1]. GBM increases in incidence from the age of 30 years and reaching its peak at 75 to 84 years old, with a median age of 65 years old [1–5]. The addition of temozolomide (TMZ) to surgical resection and radiotherapy represented the last significant advance in GBM management [6]. However, median survival remains at less than 15 months and TMZ resistance has since been reported in a proportion of tumors in which the *O*^*6*^*-methylguanine methyltransferase* (MGMT) promoter is not methylated [7–11]. In the past 40 years, only a handful of drugs have been approved for the treatment of GBM in the USA, including temozolomide (2005), bevacizumab (2009), carmustine (1996) and lomustine (1976). Overall there have been no major advances in chemotherapy for GBM in the last decade and, consequently, there is an urgent need for new treatments.

Pre-clinical animal studies form an important stage in the development and planning of new treatment designs for human clinical trials. Systematic review and meta-analysis is a powerful tool for the assessment of any treatment and is considered to be the highest level of clinical evidence by many regulatory bodies. Although systematic review and meta-analyses of clinical trials are widely reported, until recently there has been a relative paucity concerning pre-clinical literature. Groups such as the Collaborative Approach to Meta-Analysis and Review of Animal Data from Experimental Studies (CAMARADES) have undertaken systematic review and meta-analysis across a range of pre-clinical disease groups which have illustrated several important themes relevant to the design and interpretation of animal experiments [12, 13].

Previous systematic reviews of glioma model treatments have assessed the efficacy of BCNU/CCNU [14], temozolomide [15] and gene therapies [16]. In these reviews, particularly in relation to chemotherapies, results were overall concordant with human trials, although the underlying data were limited in quality and design. Several factors were found to be associated with observed treatment efficacy including factors relating to the reporting of measures to address potential risks of bias such as randomization and blinding; and those relating to study design, in particular the tumor models used.

*Tumor Suppressor Protein 53* (*TP53*) is highly associated with the regulation of topoisomerase I and II activity [17–19]. Mutations in the *TP53* gene has been found to upregulate topoisomerase II expression while leaving topoisomerase I expression unaffected [18, 20]. Topoisomerase I and II enzymes are essential in the uncoiling of supercoiled DNA to promote DNA metabolic processes. Topoisomerase I catalyse single-strand breaks that allows DNA transcription needed for protein synthesis, while Topoisomerase II catalyses the double strand breaks important for chromosome condensation. Inhibition of topoisomerases lead to the increased formation of stable topoisomerase-cleaved DNA breaks which eventually lead to cell death [21, 22]. Topoisomerase inhibition is an established strategy in oncology, and has been a target of interest in a range of different cancer types including glioma. TP53 mutations are present in the majority of primary GBMs and may contribute to tumorigenesis [23–25]. In the last 5 years, Phase I and II clinical trials evaluating the use of topoisomerase inhibitors in combination with other chemotherapeutic drugs to treat GBM have shown variable results [26–33]. In 2013, a meta-analysis performed by Leonard and Wolff [34] of data from phase I-III clinical trials from 1976 to 2011 (including 44,850 patients from 624 publications) examined the treatment efficacy of four topoisomerase inhibitors (topotecan, irinotecan, etoposide and teniposide). Of these, etoposide appeared to confer the greatest advantage in terms of increase in median survival, while others showed either no significant improvement or worsened outcome.

This variability in effects observed in clinical trials suggests that topoisomerase inhibition may have a role in treatment of glioma if the optimal conditions for efficacy (patient selection, timing of treatment, *etc*.) could be identified. This contrasts with the pre-clinical literature where the subjective impression is one of consistent efficacy. Here we describe a systematic review and meta-analysis of topoisomerase inhibition in pre-clinical GBM studies, with the aim of summarising the overall efficacy of topoisomerase inhibition in this population and whether differences in efficacy exist between different drugs and different tumour models. Furthermore, we appraise the quality of these studies and search for evidence of bias; as well as describing the variation in and impact of different study design parameters on outcome. Finally, we aim to appraise the construct validity of the models used.

## RESULTS

**Figure 1:**
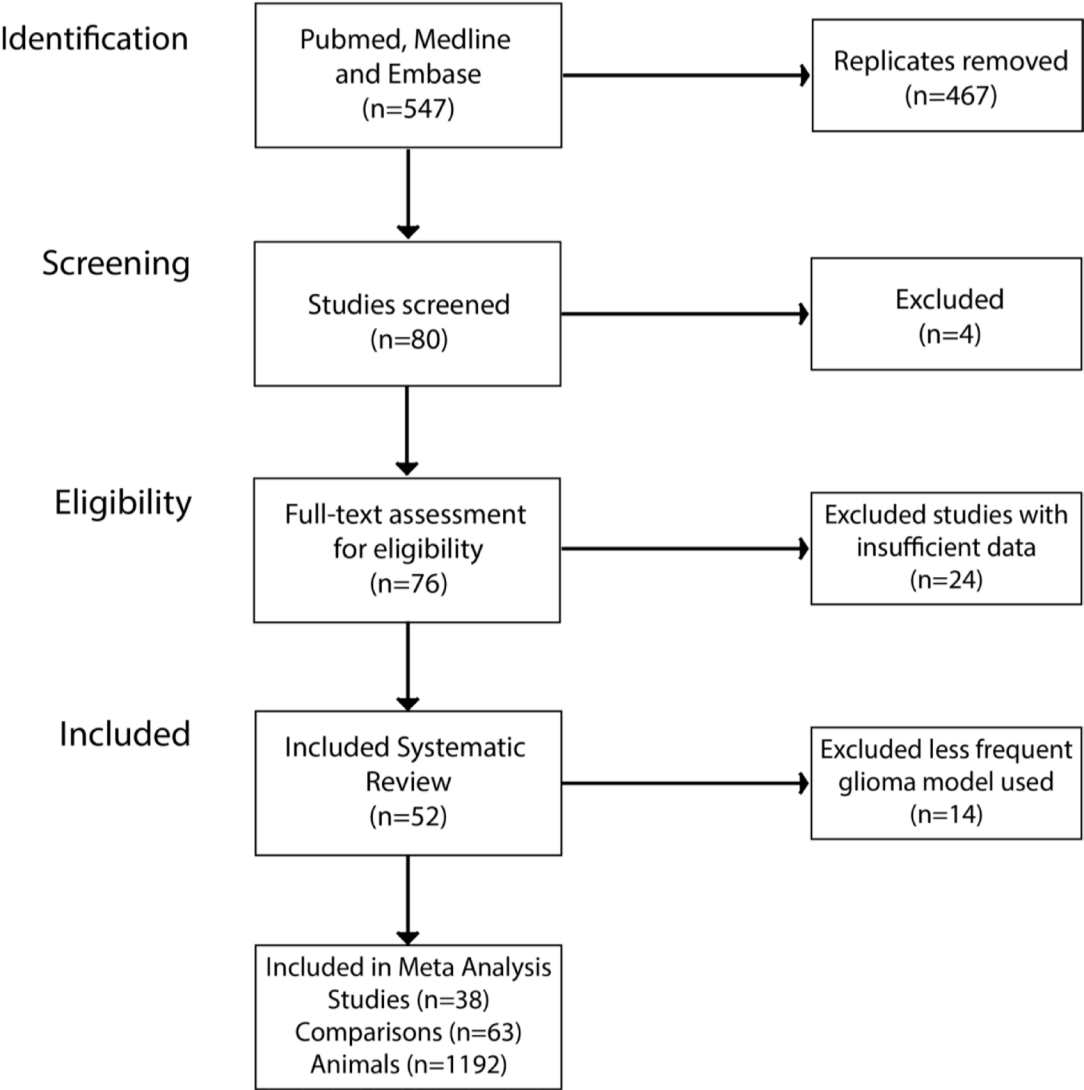
Study selection summary Three public databases were searched for keyword of interest. This returned 547 publications, after screening using our inclusion criteria we were left with 38 publications included in the meta-analysis.

After searching PubMed, Medline and EMBASE, 547 publications were identified with 76 publications satisfying the inclusion criteria (Figure 1). We excluded one study that used bioluminescent imaging to assess for change in tumor volume, as there were no direct measurements of tumor volume [35]. Other reasons for exclusion were missing or unreported data (*n* = 5), no appropriate control used for the study (*n* = 11), no measure of the variance of the volume data (*n* = 2) or the tumor volumes not reported (*n* = 2), no quantitative data reported for the outcome measure (*n* = 1), no individual drug treatment results reported (*n* = 2) and the drug being assessed in the study is not included in the list of drugs specified in the protocol (*n* = 1).

Following these exclusions, 52 publications remained reporting 61 experimental comparisons assessing animal median survival (MSR) and 29 assessing tumor volume (TVR) data; using a total of 1,611 animals. The details of the included studies are presented in Supplementary Table 1.

Of the 90 comparisons, mice were the most commonly used species for assessing both survival (*n* = 35) and tumor volume reduction (*n* = 20). Mice and rats were the only species of animal used. Comorbid animals were commonly used. Athymic animal models were most commonly observed in studies assessing survival (*n* = 26) and in studies assessing tumor volume (*n* = 20). Other comorbidities observed in the survival studies were RAG2-M (*n* = 2) and SCID (*n* = 2), which are both immunocompromised models. SCID animals were reported in two out of 29 tumor volume reduction experimental comparisons. Survival studies included 56 intracranial and five subcutaneous animal models, while tumor volume reduction studies included 11 intracranial and 18 subcutaneous animal models.

The most frequently used cell line was U87 in both survival (*n* = 14) and volume studies (*n* = 16). Other glioma models more commonly observed were U251 (*n* = 7), 9L (*n* = 8), BT4Ca (*n* = 5), GBM (*n* = 7), and 101/8 (*n* = 6). Aside from one study which used a patient-derived GBM xenograft model [36], none of the studies screened for TP53 mutation (Table 1). The majority of survival studies used xenograft models (*n* = 57), and six of the seven experiments that used ‘GBM’ models were serially passaged subcutaneous patient-derived xenografts. Most tumor models developed were orthotopic (*n* = 67).

**Table 1:** TP53 mutation status of glioma models

Cell line group	TP53 mutation status
U87	Wildtype [52]
U251	Mutant [53]
GBM	SJ – GBM2 – mutant [36]
BT4Ca	unknown
9L	Mutant [54]
101/8	Unknown

Summary of TP53 mutation status for glioma models included in this meta-analysis.

Experiments most commonly used doxorubicin (*n* = 49), followed by irinotecan (*n* = 25), etoposide (*n* = 7), topotecan (*n* = 6), and epirubicin (*n* = 3). The most frequently used route of drug delivery were intravenous (*n* = 48), intracranial (*n* = 21) and intraperitoneal (*n* = 15) administration.

### Study quality

Study quality was assessed in all 90 comparisons included using a predefined 12-item checklist (Supplementary Table 2). The study quality was modest, the median number of checklist items scored 6 out of 12 (IQR – 5–7, range 3–9). Ninety-six percent of the comparisons included in the systematic review were published in a peer-reviewed journal, 56% reported random allocation to treatment groups, 10% blinded the assessment of outcome measures, none reported sample size calculation, 88% reported compliance with animal welfare regulations, 34% stated a potential conflict of interest, 26% published “take rates”, 13% reported reasons for exclusions, 98% had a consistent site of tumor implantation, 87% reported a standard number of implanted cells in all the animals, 23% justified drug action and 62% justified the use of carriers.

### Meta-analysis

**Figure 2:**
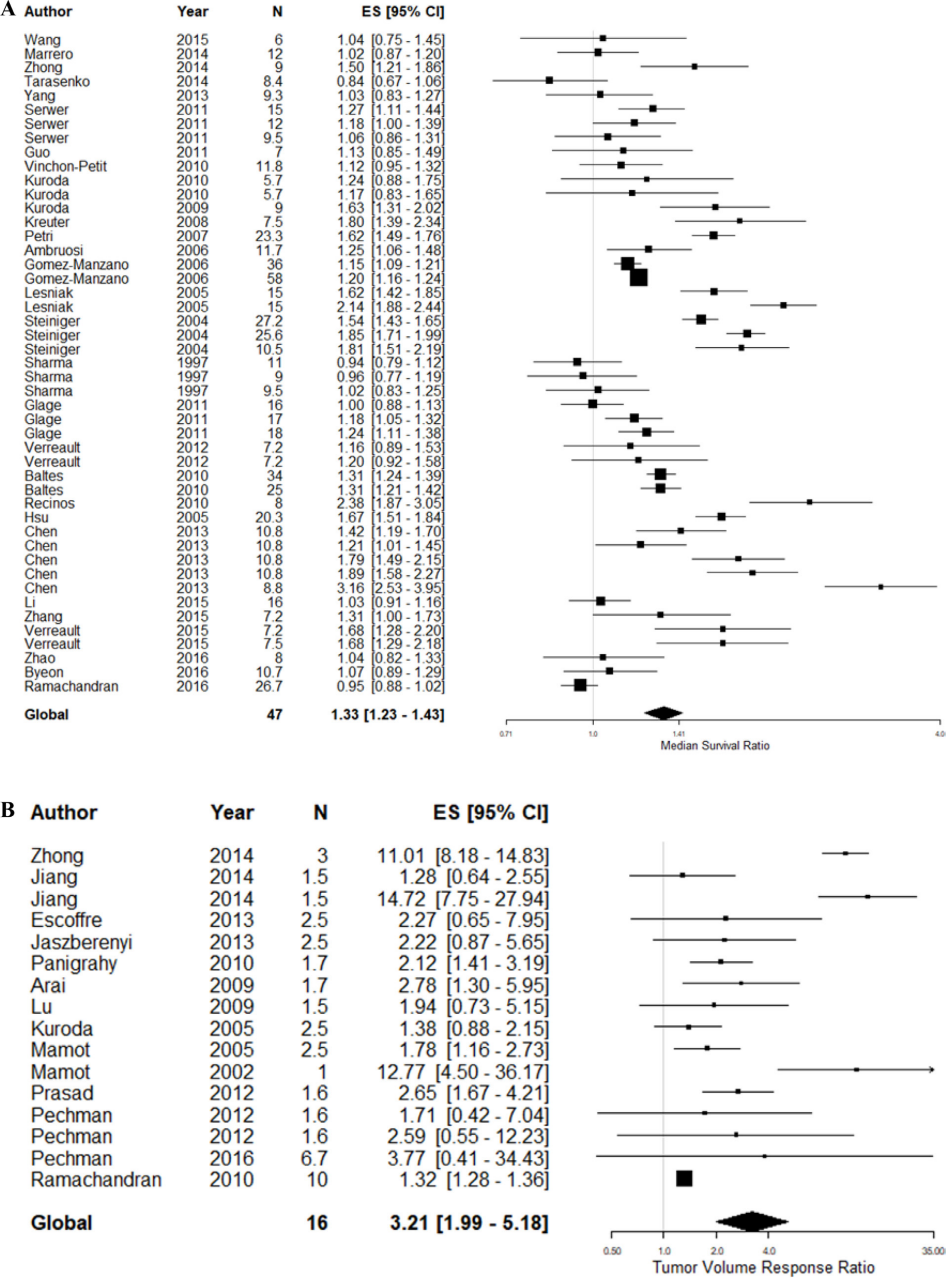
Meta-analysis of included studies based on outcome measure (**A**) Meta-analysis of studies assessing animal survival. (**B**) Meta-analysis of experimental comparisons assessing tumor volume reduction. The grey line represents neutral effect. The x-axis is shown in log scale.

In the 90 comparisons included in the systematic review, 18 different glioma models were reported (Supplementary Table 1). However, previous meta-analyses in this field have suggested a substantial degree of covariance between study design parameters, particularly the choice of tumor model [15, 16]. To limit this covariance, we proceeded to meta-analyse studies reporting experiments using glioma models which had been used in five or more studies reporting the corresponding outcome measure. This involved 47 survival and 16 tumor volume experimental comparisons using a total of 1,192 animals (Figure 2).

Overall, animals bearing gliomas treated with a topoisomerase inhibitor survived 1.32 times (95% CI: 1.23 – 1.43) longer than control animals, substantial between-study heterogeneity was observed (*F* = 7.1, *p* < 0.001, *I*^2^ = 92.2%). On the other hand, topoisomerase inhibition reduced tumor growth by 69% (TVR response ratio: 3.2; 95% CI: 1.99 – 5.18) compared to control animals, substantial between-study heterogeneity was also observed in this dataset (*F* = 4.78, *p* < 0.001, *I*^2^ = 95.5%).

### Meta-regression

Heterogeneity was high in both datasets, warranting further analysis to identify study quality or design items associated with treatment efficacy. As specified in our protocol, we used a multivariable meta-regression, designed to identify and account for covariance between study characteristics which is common in heterogeneous datasets. In this instance, we suspected glioma model selection to be a frequent confounder in univariable analyses.

We initially performed univariable meta-regression to assess the impact of study characteristics on both survival and tumor volume. Variables tested included the glioma model, animal species, site of tumor cell implantation, drug used, dosage, type of control used, route of drug delivery, frequency of drug administration, randomisation, blinding of outcome assessment and quality score. We deemed the data sufficient to proceed to multivariable meta-regression with the survival dataset (47 comparisons) but not the tumor volume dataset (18 comparisons).

For the multivariable meta-regression model, based on our previous experience, we allowed inclusion of one variable for every ten comparisons – thereby permitting input of five predictors. We selected these variables *a priori* to be glioma model, site of implantation, drug used, route of delivery, quality score and type of control used for the experiment. However, because majority of the tumors were orthotopic (*n* = 44/47) we did not include site of implantation, instead we included the variable which had returned the largest *F*-value in the univariable analysis, that being the nature of the control used. Following the meta-regression, each predictor was tested *post-hoc* with a Wald test – with a significant result implying an independent predictive value of the variable of interest.

Our analysis of interest was the multivariable model; we report these data first. Where this was not possible, for remaining predictors in survival dataset and for the tumor volume dataset, we report results from the univariable meta-regression.

### Survival dataset

The multivariable meta-regression model was significantly associated with median survival outcome, suggesting a predictive value of at least one variable offered (*F* = 6.08, *p* < 0.0001), and residual *I*^2^ of 74.24%. When tested with a Wald test, 4 of 5 variables offered to the model (glioma model, drug, type of control, and route of drug delivery) were independently associated with heterogeneity in the survival dataset (Table 2).

**Table 2:** Multivariable meta-regression

Variables	*F*	*p*
Model	6.08	0.0005^*^
Type of control	3.85	0.0185^*^
Route of drug delivery	8.61	0.001^*^
Drug	3.74	0.0207^*^
Quality score	0.91	0.3482

Multivariable meta-regression of predictors identified a priori.

Legend: ^*^p-value < 0.05.

**Figure 3:**
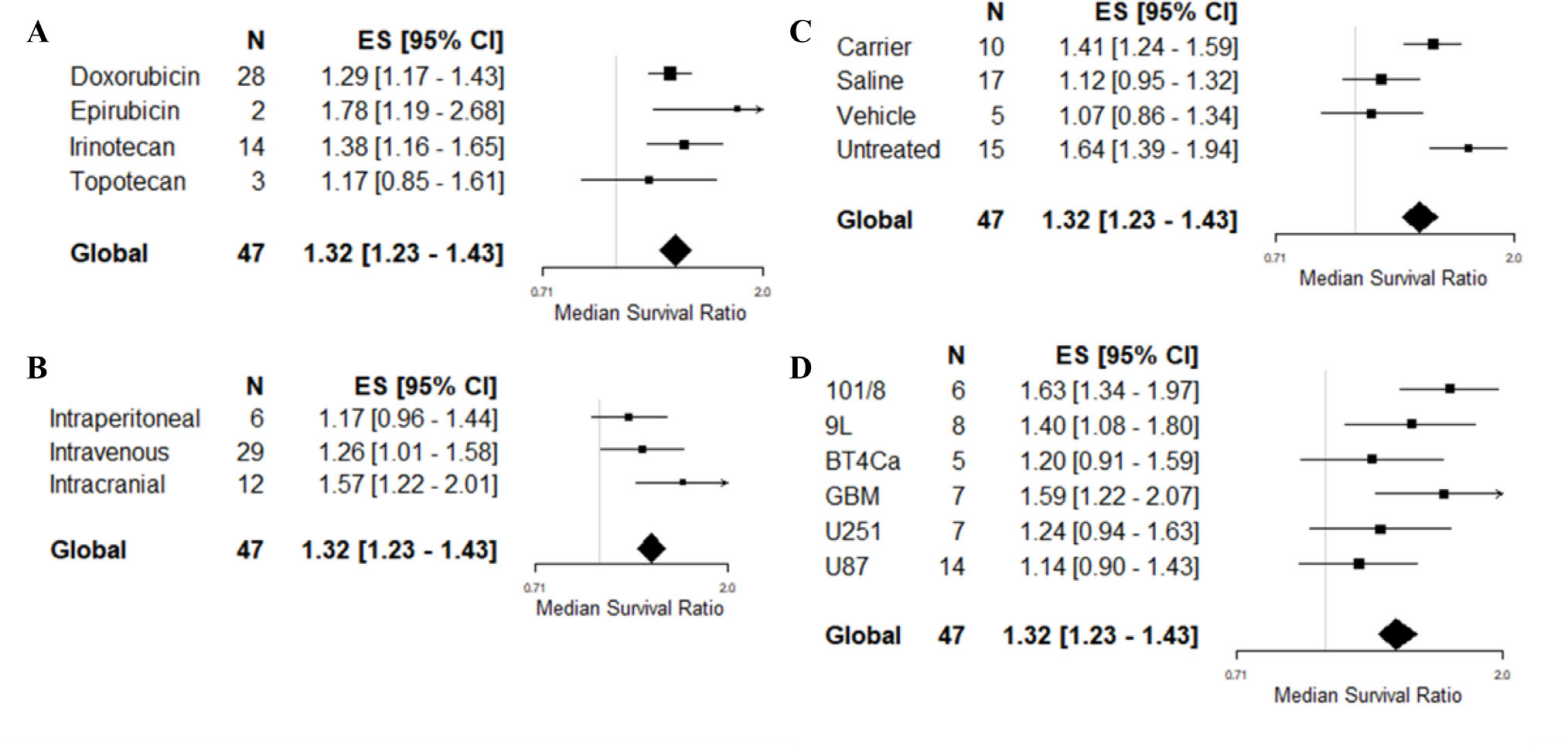
Multivariable meta-regression based on survival outcome Forest plots of (**A**) choice of drug, (**B**) route of drug delivery, (**C**) type of control and, (**D**) choice of glioma model for the study. Median survival ratio was calculated by dividing the mean outcome of the treatment groups by the mean outcome of the control group. The solid grey line represents neutral effect. The x-axis is shown in log-scale.

All 4 drugs used appeared to be associated with treatment efficacy (Figure 3A), with the most commonly used drugs (doxorubicin and irinotecan) associated with similar outcomes (MSR: 1.29, 95% CI 1.17–1.43 and 1.38, 1.16–1.65, respectively). Epirubicin, used in only 2 experiments, was associated with greater efficacy (1.78, 95% CI 1.19–2.68). The choice of drug was independently predictive of treatment outcome in the multivariable model (*F* = 3.74, *p* < 0.05), although not in the univariable model (*F* = 1.14, *p* > 0.0042). The route of drug delivery was associated with survival outcome in the multivariable model (*F* = 8.61, *p* < 0.05, Figure 3B), with intracranial treatment appearing to be associated with greater efficacy than systemic routes. There was no association seen on univariable analysis (*F* = 3.92, *p* > 0.0042). Drug dose for doxorubicin (*F* = 0.01, *p* > 0.0042) and irinotecan (*F* = 0.7, *p* > 0.0042) was not significantly associated to an improved survival outcome in the univariable analysis. Drug dose for epirubicin and topotecan were not assessed due to low number included in the meta-analysis. The type of control, included into the multivariable analysis because of a large *F*-value on univariable analysis (*F* = 9.94, *p* < 0.0042), was also predictive of outcome on multivariable meta-regression (*F* = 3.85, *p* < 0.05). Studies where the control was untreated or given a carrier were associated with greater efficacy than those controlled with vehicle or saline (Figure 3C).

All 6 glioma models used in survival experiments were associated with treatment efficacy (Figure 3D). The choice of glioma model was associated with outcome heterogeneity on multivariable meta-regression (*F* = 6.08, *p* < 0.05), with human GBM (1.58, 1.22–2.07) and rat 101/8 (1.63, 1.34–1.97) cell lines associated with greater efficacy than the most frequently reported U87 cell line (1.14, 0.90–1.43). Tumor model selection was not associated with heterogeneity on univariable meta-regression (*F* = 3.08, *p* > 0.0042). There were no associations between total study quality score and survival outcome on either multivariable (*F* = 0.91, *p* > 0.05) or univariable (*F* = 0.18, *p* > 0.0042) analysis.

**Figure 4:**
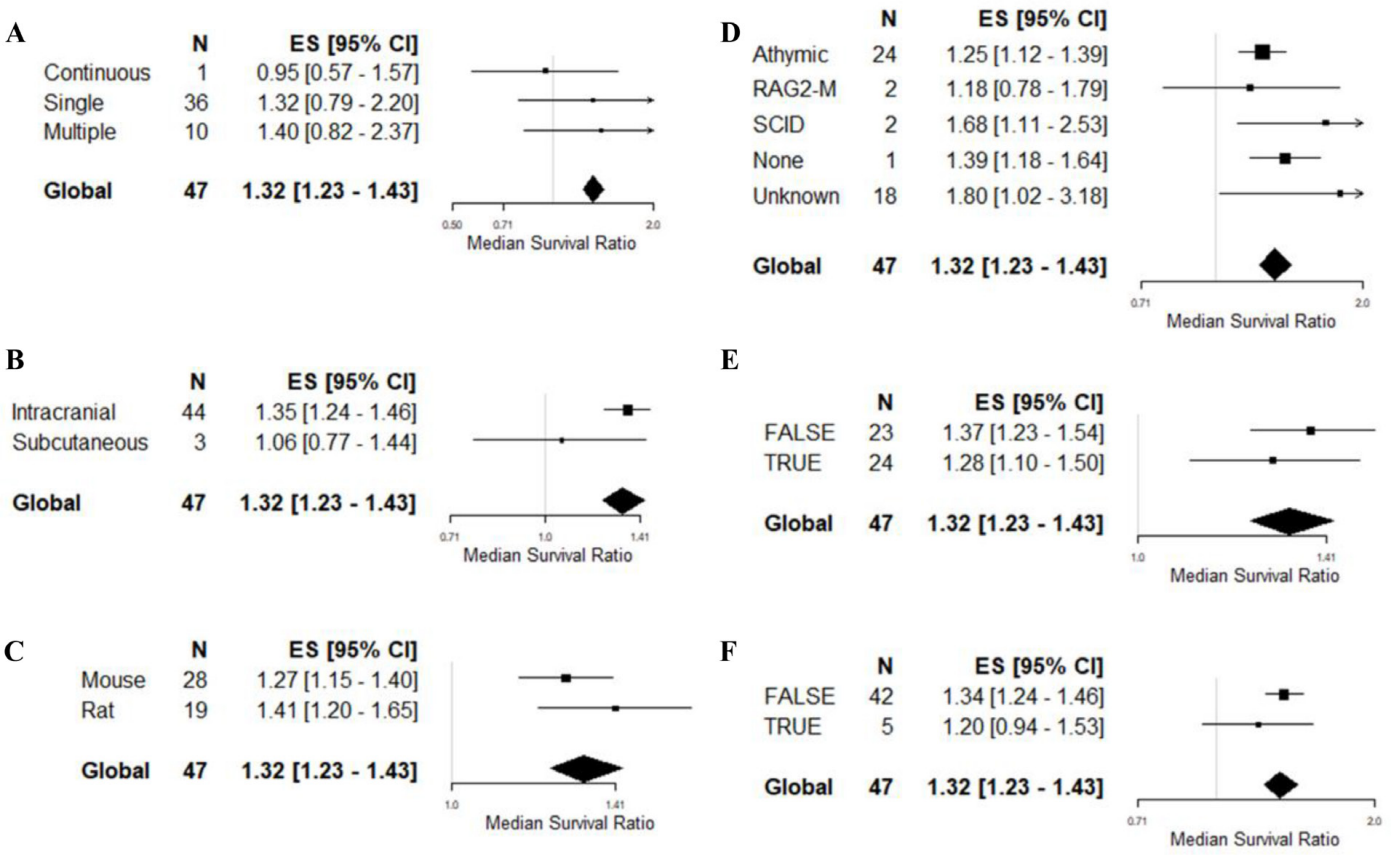
Univariable meta-regression based on survival outcome Forest plots of (**A**) frequency of drug administration, (**B**) tumor site implantation, (**C**) animal species, (**D**) comorbidity, (**E**) randomisation, and (**F**) blinding of outcome assessment. The solid grey line represents neutral effect. The x-axis is shown in log-scale.

The remaining variables were tested on univariable meta-regression only (Table 3, Figure 4). There were no associations between survival outcome and frequency of drug administration (*F* = 1.68, *p* > 0.0042), site of tumor implantation (*F* = 2.32, *p* > 0.0042), species (*F* = 1.68, *p* > 0.0042), comorbidity (1.14, *p* > 0.0042), the reporting of randomised group allocation (*F* = 0.79, *p* > 0.0042) or blinded assessment of outcome (*F* = 0.79, *p* > 0.0042).

**Table 3:** Univariable meta-regression

	Survival		Tumor volume	
Variables	*F*	*p*	*F*	*p*
Animal species	1.68	0.2011	−	−
Model	3.05	0.0198	na	na
Comorbidity	1.14	0.3526	−	−
Site of tumor implantation	2.32	0.1349	2.47	0.1386
Drug	1.14	0.3448	1.05	0.405
Route of drug delivery	3.92	0.0271	1.17	0.3601
Type of control	9.94	<0.0001^*^	0.29	0.8779
Frequency of administration	1.07	0.3518	0.21	0.8168
Randomisation	0.79	0.3801	0.06	0.8146
Blinded outcome assessment	0.79	0.3785	na	na
Quality score	0.18	0.671	0.81	0.691

Comparison of univariable meta-regression for survival and tumor volume experimental comparisons.

Legend: ^*^p-value < 0.05; na - not applicable.

### Tumor volume dataset

**Figure 5:**
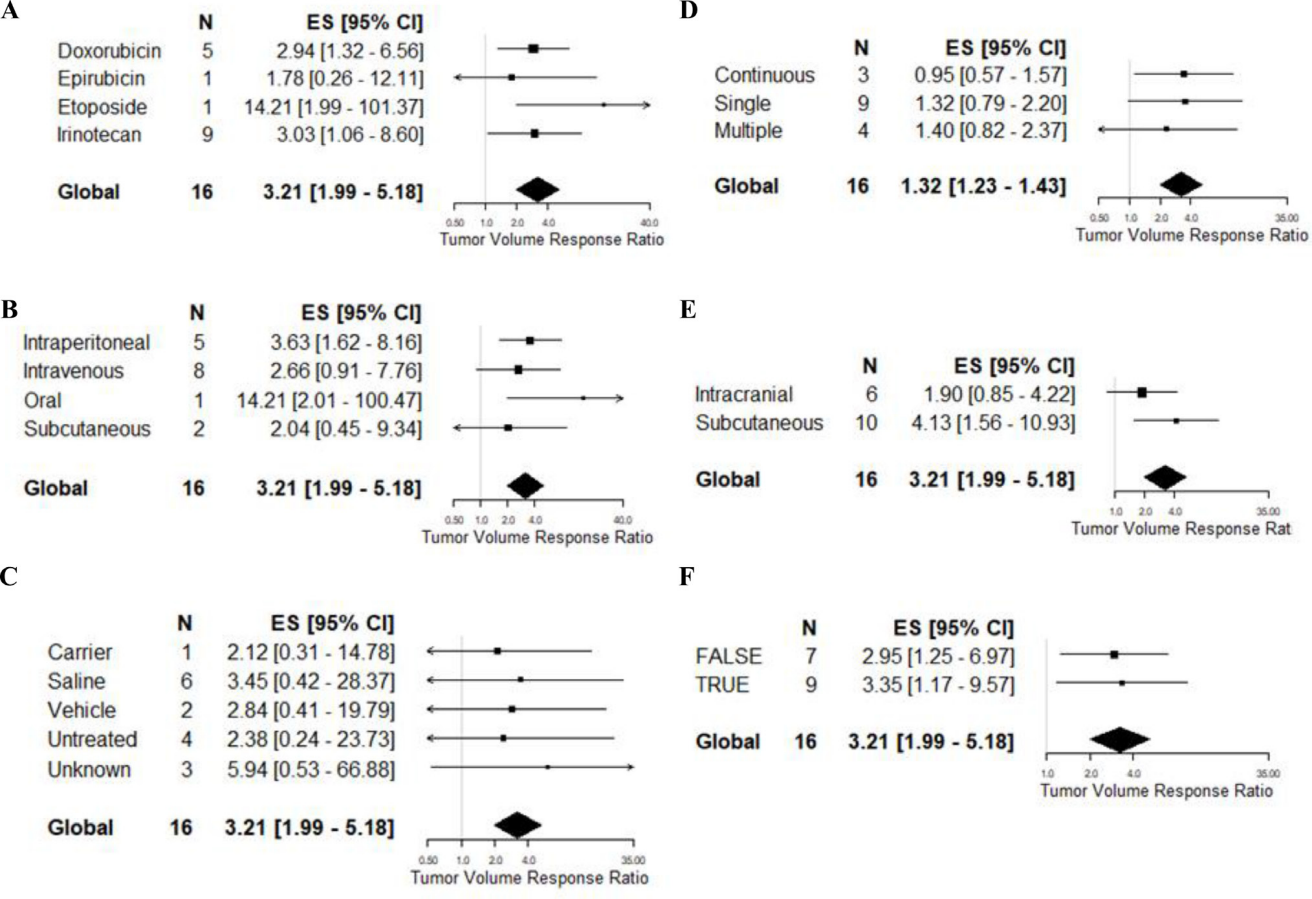
Univariable meta-regression based on tumour volume reduction Forest plots of (**A**) drug of choice, (**B**) route of administration, (**C**) type of carrier, (**D**) frequency of administration, (**E**) site of tumor implantation, and (**F**) randomisation. The solid grey line represents neutral effect. The x-axis is shown in log-scale.

As there were only 16 experiments included in this meta-analysis, we did not apply multivariate meta-regression. All 16 experiments used a U87 glioma model reported and therefore we could not apply glioma model to a univariable meta-regression. Similarly, the majority of studies used mice (13/16) and athymic animals (14/16), and all were not blinded. Consequently, we did not test species, comorbidity or blinded assessment of outcome. None of the remaining variables, when applied to univariable meta-regression (Table 3, Figure 5), were found to be predictive of volume outcome, including drug (*F* = 1.05, *p* > 0.0056), doxorubicin dose (*F* = 0.24, *p* > 0.0056), irinotecan dose (*F* = 0.04, *p* > 0.0056), route of delivery (*F* = 1.17, *p* > 0.0056), type of control (*F* = 0.29, *p* > 0.0056), frequency of drug administration (*F* = 0.21, *p* > 0.0056), site of tumor implantation (*F* = 2.47, *p* > 0.0056), randomised group allocation (*F* = 0.06, *p* > 0.0056) and quality score (*F* = 0.81, *p* > 0.0056).

### Post-hoc assessment of drug carriers

Two of the significant results from the two analyses pertained to drug delivery – by way of route of delivery directly and indirectly in the way of the type of control used. We have observed variance in the use of drug carriers already and it is possible that the use and choice of carriers could affect both of these significant results. As such, we thought it important to describe the frequency of carrier use (Supplementary Table 3), and have subsequently included this stratification *post-hoc* in the univariable metaregression.

**Figure 6:**
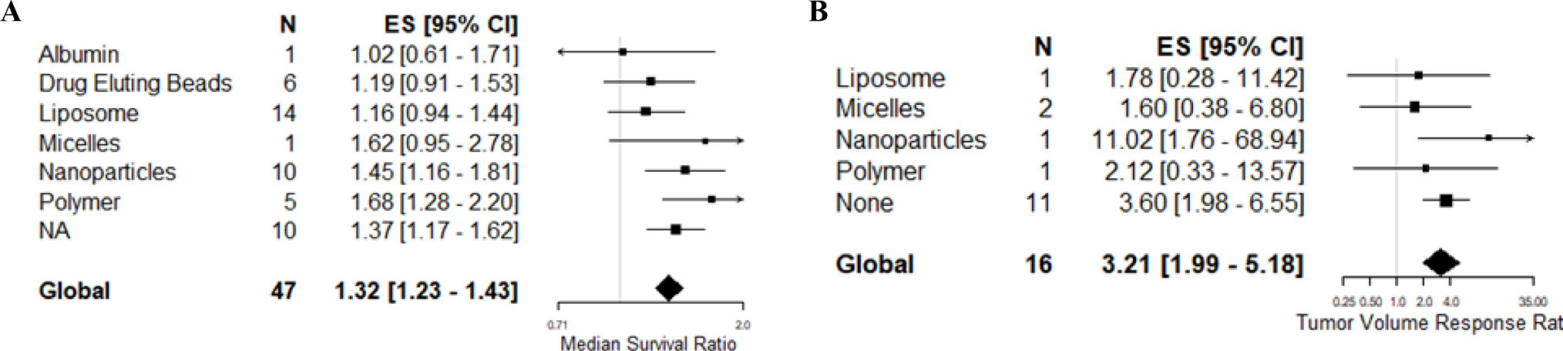
Post-hoc univariable meta-regression analysis of choice of carriers Post-hoc univariable analysis of carriers used in studies assessing (**A**) survival and (**B**) tumor volume reduction.

Of the 52 publications included in the systematic review, 58% (*n* = 30) used a carrier for drug delivery. The most frequently reported carriers were nanoparticles (*n* = 8) and liposomes (*n* = 7) as drug carriers all of which were administer intravenously except for one study using nanoparticles that administered via intraperitoneal route. Other carriers observed were polymers (*n* = 6), drug eluting beads (*n* = 3), nanoliposomes (*n* = 2), albumin (*n* = 1), micelles (*n* = 1), microbubbles (*n* = 1), and microspheres (*n* = 1). Of the 38 publications included in the meta-analysis, 25 used a carrier intended to enhance drug delivery into the brain. The choice of carrier was not associated with heterogeneity in the survival (*F* = 2.04, *p* = 0.0824) or volume (*F* = 0.95, *p* = 0.472) datasets on univariable meta-regression (Figure 6).

### Publication bias

**Figure 7:**
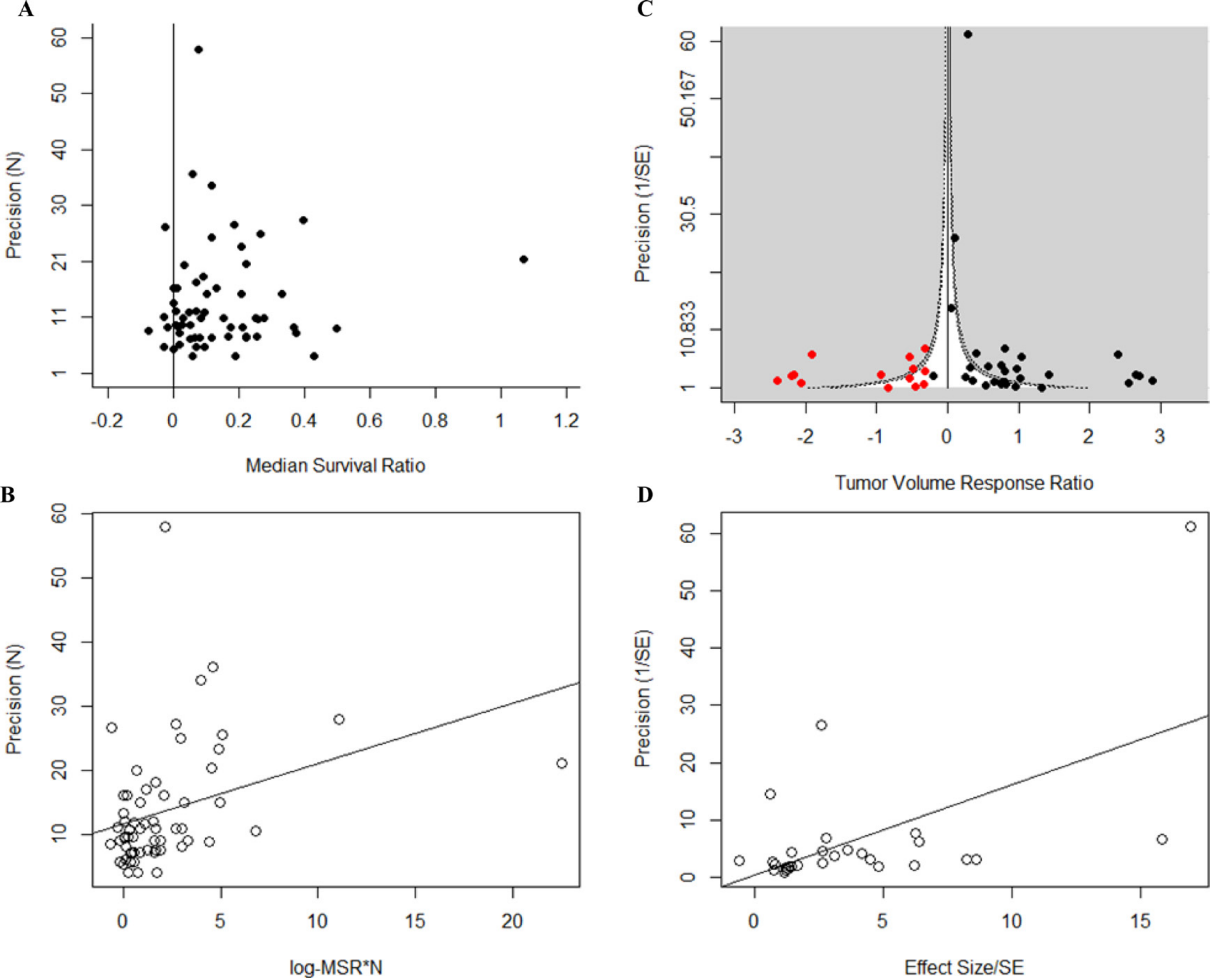
Publication bias Funnel plots and Egger’s regression publication bias plot for all studies included in the systematic review for both (**A** and **B**) survival and (**C** and **D**) tumor volume datasets. Survival studies show significant publication bias (*p* < 0.001) as shown by the asymmetry in (A) and by means of a positive intercept (11.24) in (B). For the tumor volume studies, publication bias was observed by means of funnel plot asymmetry observed in (C). Trim and fill imputed 14 ‘missing’ studies (red dots) thus reducing overall efficacy estimate in (C). Egger’s regression analysis of the tumor volume dataset did not return a significantly positive intercept (D). Funnel plots show the effect size (x-axis) against study precision (y-axis). Egger’s regression publication bias plot shows the standardised effects size (x-axis) against study precision (y-axis). Solid black lines in (A) and (B) represent the level of neutral effect. Funnel plot in (C) is contour-enhanced. The light grey shading represents area of high statistical significance, while the white area represents the area of non-statistical significance. The darker shaded contour line represents the area of associated statistical significance (*p* = 0.05).

Taking all studies included in the systematic review, we found evidence of publication bias in both datasets. For the survival dataset, publication bias was observed by means of an asymmetric funnel plot (Figure 7A), and a significantly positive intercept in the Egger’s regression test (*B* = 11.24, *t* = 8.67, *p* < 0.001, Figure 7B). We observed dramatic funnel plot asymmetry in the volume dataset (Figure 7C), although Egger’s regression test did not show a significantly positive intercept (*B* = 0.377, *t* = 0.151, *p* > 0.05, Figure 7D). Trim and Fill analysis suggested the presence of 14 ‘missing’ studies in the tumor volume dataset (Figure 7C): addition of the ‘missing’ studies dramatically reduced overall efficacy from 2.35 to 1.35. We applied a contour overlay to the funnel plot (Figure 7C) to help identify the cause of funnel plot asymmetry [37]. In this case, the imputed ‘missing’ tumor volume studies are equally distributed between the areas of high and non-statistical significance.

## DISCUSSION

In this study, we report the efficacy of five different drugs - doxorubicin, epirubicin, etoposide, irinotecan and topotecan – in pre-clinical glioma models. Fifty-two publications (90 comparisons) satisfied our inclusion criteria. Of these, 38 publications with 63 experimental comparisons consisting of 1,192 animals for treatment efficacy was used for the meta-analysis. Study design was heterogeneous and the risk of bias was high.

Nonetheless, we observed that efficacy estimates favoured treatment in all drugs in both survival and tumor volume data. We have applied a multivariable meta-regression which suggests that glioma model, drug choice, route of drug delivery and type of control are each independently associated with survival outcome in this preclinical setting.

### Validity of statistical approach

In previous reviews of the glioma literature [14–16], a large number of significant results on stratified meta-analysis and a particularly large range of efficacies observed across a number of glioma models has generated suspicion of collinearity and, as such, rendered results difficult to interpret.

We therefore devised two strategies to counter this: firstly, to exclude infrequently reported glioma models (those reported in fewer than five experiments), and secondly to apply multivariable meta-regression to the remaining data following a standard (univariable) meta-regression. The univariable meta-regression returned fewer significant findings than previous stratified meta-analyses in glioma studies, probably because meta-regression is more conservative than stratified meta-analysis [38, 39]; and because we included fewer studies. Based on the multivariable meta-regression analysis, four variables were independently associated with survival heterogeneity. Three of these, namely glioma model, drug and route of drug delivery were nonsignificant on univariable meta-regression. The revelation of these associations on multivariable modelling confirms the presence of collinearity in this data as suspected, and thus validates multivariable modelling in this context.

We suggested that the selection of carrier may have had confounded analyses of route of delivery and control used, both of which returned significant results meta-regression, and on this basis, we felt it appropriate to include carrier in univariable meta-regression *post hoc*. While this result was non-significant, we did not include in a multivariable model and so it remains possible that a confounding effect of drug carriers may exist. We observed no significant associations on the univariable analysis of the tumor volume dataset, and this is likely a manifestation of a large degree of heterogeneity, collinearity and small sample size.

### Impact of factors relating to study quality

On further inspection of the included studies we found a large range of experimental design, relating to both design parameters and measures to reduce the risk of bias. Randomisation and blinded assessment of outcome are important measures of overall study quality and inflated treatment efficacy in the context of a lack thereof is a phenomenon well-described in the pre-clinical neurosciences literature [40] and in previous glioma studies [15, 16]. Infrequent reporting of randomization and blinding in this dataset is consistent with other studies although we have not observed an association with experimental outcome. This may be because of collinearity or small sample size, and might be revealed in a larger multivariable meta-regression of glioma studies.

Furthermore, we have found evidence of publication bias in both datasets by way of asymmetrical Funnel plots and a positive Egger’s regression intercept in the survival dataset and ‘missing studies’ found in Trim and Fill analysis of the tumor volume dataset. These suggest that there is a relative lack of small, inefficacious studies that are not reported for several reasons [41] and resulting in a consequent inflation of efficacy perceived both in subjective impressions of the literature and in this meta-analysis. Contour enhancement of the funnel plot suggests that the asymmetry observed in the tumor volume dataset may also be due to other confounding factors rather than publication bias alone.

### Factors relating to study design

Animal experiments may often be viewed as a stepping stone from bench to bedside, given the innate similarities between both hosts and their diseases – in physiological and genetic terms. Unfortunately, animal models have several key features that, although not preclusive of their value in research, render them imperfect.

Animal experiments are often designed to minimize variables and thus tend to report the use of genetically similar individuals seeded with tumors derived from a single cell line. Immortalized cell lines used to produce xenograft models are well established and easy to cultivate, although have several key limitations. An example is the most commonly used glioma xenograft – U87 – which has been observed to have a distinct DNA profile and grow in a pattern not typical of a human GBM [42–44]. They are genetically homogenous and behave predictably – thus lacking heterogeneity observed in GBM patients. Pooling of studies in meta-analysis provides a way to simulate this heterogeneity and retest a treatment in this context. However, its value is dependent on the underpinning data – in terms of quality and the experiment’s ability to replicate the biology and heterogeneity of human disease. An example of note in this context is the *TP53* status of tumour model used in the studies in this analysis. Of the six GBM models found in 70 experimental comparisons included in the meta-analysis, four have known *TP53* mutation status but only one study screened for this. Based on trend, better response can be observed on *TP53* wildtype cell lines (Table 1, Figure 3D). We have shown that glioma model selection is one of the most important predictors of treatment efficacy; more so than features such as drug of choice and several quality parameters.

A discrepancy we have observed between this meta-analysis and a similar review of human clinical trials is the absence of doxorubicin [34], contrasting with animal studies in which it was the most commonly used drug. Glioma model selection should be considered very carefully in the design phase of future studies to improve translation of pre-clinical investigations to human clinical use.

In this study we have not pursued the effects of the large range of dosing schedules used. Our primary intention was to review the overall efficacy of topoisomerase inhibitors rather than to tease out the intricacies of dose scheduling. Furthermore, to do this would require a larger dataset to permit analysis of timing and duration of treatment, dosage, frequency and number of cycles with sufficient power. To provide a rough insight we used total dose, which was not associated with heterogeneity for doxorubicin or irinotecan in either dataset.

### Limitations of this review

We believe the conclusions drawn from this study are important, however these should be taken with caution due to a number of limitations. Although meta-analysis is a powerful tool, it comes with its weaknesses. It is at the mercy of the rigour in which it is undertaken, and we have minimised this through transparent reporting and prior publication of a protocol. Nonetheless, our search terms only included generic drug names (e.g. irinotecan). In omitting older synonyms, such as CPT-11, we may have missed a small number of older studies. The findings of a meta-analysis are only as reliable as the primary data included – we found that quality was overall low, which should be acknowledged when interpreting the results of this study. Meta-analysis is a tool to observe associations between study design parameters and efficacy; these findings do not imply causality, so should only be considered as hypothesis-generating. While we have identified and attempted correction for a number of confounding factors, further unacknowledged confounders could still have distorted our results. An example of this is the carrier used for drug delivery, which we did not prespecify as a variable of interest in our protocol. When a confounding effect subsequently became mechanistically and statistically plausible, we were reluctant to include in the multivariable model in the interest of preserving the integrity of our analysis. Findings and questions arising from this review should be answered with prospective, high-quality studies.

## CONCLUSIONS

Topoisomerase inhibition has failed to prove successful in clinical trials despite apparently consistent efficacy in preclinical studies. However, a number of concerns arise relating to the internal and construct validity of the animal literature: the existing literature is at high risk of bias, with evidence of augmented perceived treatment efficacy, and animal research has limitations in the recapitulation of human disease. Nonetheless, factors such as glioma model, type of control used, route of administration and type of drug used appear to predict outcome and must be taken into consideration when planning future studies. We believe that further high-quality, prospective *in vivo* studies accommodating these conclusions would be invaluable in helping to further define the role for topoisomerase inhibition in clinical GBM.

## MATERIALS AND METHODS

### Experimental design

We aimed to undertake a systematic review and meta-analysis of topoisomerase inhibition in preclinical glioma models; outcome measures of interest were changes in survival and tumour volume. Furthermore, we anticipated significant heterogeneity between studies, which we investigated using meta-regression, and publication bias, which we have investigated using funnel plots, Egger’s regression test and Trim and Fill analysis.

This was conducted as described in a protocol, published online on 15 October 2015. Available at http://www.dcn.ed.ac.uk/camarades/research.html#protocols.

### Sources

A literature search of phase II clinical trials revealed that doxorubicin, epirubicin, etoposide, irinotecan and topotecan are the most commonly used FDA-approved topoisomerase inhibitors for the treatment of GBM. In August 2014, we searched PubMed, Medline and Embase for the following keywords: (glioblastoma or glioblastoma multiforme or GBM or high-grade glioma) AND (Doxorubicin OR Epirubicin OR Etoposide OR Irinotecan OR Topotecan). The search was limited to *in vivo* studies with predeveloped filters [45, 46], with no language or publication date restrictions. The search was updated in July 2016.

### Inclusion criteria

During the screening process, criteria for inclusion required the following information from the studies: (i) a topoisomerase inhibitor used as monotherapy, (ii) use of an adult high-grade glioma model, (iii) intracranial or subcutaneous tumor implantation and (iv) tumor volume or median survival reported as the outcome measure. The title and abstracts were screened by two of the authors (TJ and TH) for inclusion.

### Data extraction

All data extracted were entered into the CAMARADES data manager application. Data were extracted regarding the publication (author names, year of publication, and title), intervention (drug used, dose, dose frequency, route of administration, drug delivery method, delay to treatment, and time of assessment based on day 0 of treatment), animal population (species, strain, sex, age, and number of animals per group), tumor implantation (cell type used, site of implantation, number or volume of cells inoculated and inoculation method used) and outcome measures (tumor volume and median survival data). All data used in this study is available in Supplementary Dataset 1.

The median survival and tumor volume data were estimated from graphs using a desktop ruler when these were not provided in the text.

### Study quality scoring

A modified 12-item checklist adapted from previously published studies was used to assess the quality of the studies and determine possible publication bias (Supplementary Table 2) [14–16].

### Statistical analysis

A summary statistic was initially performed for each individual study. For survival data, we used median survival ratio as described previously [47] and for tumor volume data we used response ratio [48]. Data were then normalized via log transformation and pooled using DerSimonian and Laird random-effects meta-analysis. We weighted survival studies by the number of animals in the study as a surrogate marker for inverse variance [47].

We assessed for the presence of heterogeneity using the *I*^2^ statistic and investigated sources of heterogeneity using meta-regression (via the *metareg* command in STATA 12.0). To limit the covariance observed in glioma models, we excluded those studies using glioma models reported in fewer than five experiments. We then performed a univariable meta-regression followed by a multivariable meta-regression where sufficient data were present; further details are described in the results section. Bonferroni correction was applied on the comparisons in the univariable meta-regression; critical *p*-value was adjusted to *p* = 0.0042 for survival data (12 comparisons) and *p* = 0.0056 for volume data (nine comparisons). In multivariable meta-regression, critical *p*-value was set at 0.05. Publication bias was assessed using contour-enhanced funnel plots, Egger’s regression test and ‘Trim and Fill’ analysis using ‘foresplot’ and ‘metafor’ R packages [37, 49–51]. Contour enhancement of funnel plots was performed to help identify cause of funnel plot asymmetry [37]. All tests reported are two-sided.

## SUPPLEMENTARY MATERIALS TABLES








