# Supplementary material for: A systematic review and meta-analysis of topoisomerase inhibition in pre-clinical glioma models
Source: Oncotarget. 2018 Jan 29;9(13):11387–401. doi: 10.18632/oncotarget.24334 (PMC5834287; doi:10.18632/oncotarget.24334)
Supplement: Supplementary file 1 [file oncotarget-09-11387-s001.pdf]

A systematic review and meta-analysis of topoisomerase inhibition in pre-clinical glioma models

SUPPLEMENTARY MATERIALS

Supplementary Table 1: Study characteristics-contains a summary of all the study characteristics extracted from all the references included in the study. See Supplementary_Table_1

Supplementary Table 2: Study quality scores-contains the study quality scores of each study according to a 12-item checklist to determine publication bias. See Supplementary_Table_2

Supplementary Table 3: Carrier types-contains a list of the types of carriers observed in the studies included in this study

First Author	Year	Outcome Measure	Route of admin	Type of carrier
Marrero, L.	2014	Survival	IV	albumin
Zhong, Y.	2014	Survival	IV	nanoparticles
Zhong, Y.	2014	Volume	IV	nanoparticles
Yang, Y.	2013	Survival	IV	liposome
Serwer, L.	2011	Survival	IV	nanoliposome
Serwer, L.	2011	Survival	IV	nanoliposome
Serwer, L.	2011	Survival	IV	nanoliposome
Guo, L.	2011	Survival	IV	liposome
Vinchon-Petit, S.	2011	Survival	IV	drug eluting beads
Arai, T.	2010	Volume	SC	polymer
Kuroda, J.	2009	Survival	IV	micelles
Kuroda, J.	2009	Volume	IV	micelles
Kreuter, J.	2009	Survival	IV	nanoparticles
Petri, B.	2007	Survival	IV	nanoparticles
Ambruosi, A.	2006	Survival	IV	nanoparticles
Gomez-Manzano, C.	2006	Survival	IP	nanoparticles
Gomez-Manzano, C.	2006	Survival	IP	nanoparticles
Mamot, C.	2005	Volume	IV	liposome
Mamot, C.	2005	Volume	IV	liposome
Lesniak, M.	2005	Survival	IC	polymer
Lesniak, M.	2005	Survival	IC	polymer
Steiniger, S.	2004	Survival	IV	nanoparticles
Steiniger, S.	2004	Survival	IV	nanoparticles
Steiniger, S.	2004	Survival	IV	nanoparticles
Sharma, U.	1997	Survival	IV	liposome
Sharma, U.	1997	Survival	IV	liposome
Sharma, U.	1997	Survival	IV	liposome
Glage, S.	2011	Survival	IC	drug eluting beads
Glage, S.	2011	Survival	IC	drug eluting beads
Glage, S.	2011	Survival	IC	drug eluting beads
Verreault, M.	2012	Survival	IV	liposome
Verreault, M.	2012	Survival	IV	liposome
Baltes, S.	2010	Survival	IC	drug eluting beads
Baltes, S.	2010	Survival	IC	drug eluting beads
Recinos, V.	2010	Survival	IP	polymer
Hsu, W.	2005	Survival	IC	polymer
Li, J.	2015	Survival	IV	polymer
Zhang, C.	2015	Survival	IV	liposome
Zhao, Y.	2016	Survival	IV	liposome
Byeon, H.	2016	Survival	IV	nanoparticles

Supplementary Dataset 1: Raw data for the systematic review and meta-analysis of topoisomerase inhibition in pre-clinical glioma models-includes extracted data and analysis results acquired from articles included in the systematic review. Data management was performed using the CAMARADES data manager (Access database). Specific contents per excel sheet: i) Raw data, ii) Study quality, iii) Survival analysis, iv) Survival publication bias (pubbias), v) Tumor volume analysis, and vi) Tumor volume publication bias (pubbias). See Supplementary_Dataset_1
