# Supplementary material for: A systematic review and meta-analysis of topoisomerase inhibition in pre-clinical glioma models
Source: Oncotarget. 2018 Jan 29;9(13):11387–401. doi: 10.18632/oncotarget.24334 (PMC5834287; doi:10.18632/oncotarget.24334)
Supplement: Supplementary file 2 [file oncotarget-09-11387-s002.docx]

**Supplementary Table 1: Study characteristics**

| **Name** | **Year** | **Quality score** | **Total N** | **Animal** | **Comorbidity** | **Broad glioma model** | **Drug** | **Broad Route of Delivery** | **Single/ Multiple** | **Type of Control** | **Type of Carrier used** | **Outcome Measure** |
| --- | --- | --- | --- | --- | --- | --- | --- | --- | --- | --- | --- | --- |
| Wang, W. | 2015 | 6 | 6.00 | Mouse | Athymic | U251 | Irinotecan | IP | Multiple | Vehicle | NA | Median Survival |
| Marrero, L. | 2014 | 9 | 12.00 | Mouse | Athymic | U87 | Doxorubicin | IV | Multiple | Vehicle | Albumin | Median Survival |
| Zhong, Y. | 2014 | 5 | 9.00 | Mouse | Athymic | U87 | Doxorubicin | IV | Multiple | Saline | Nanoparticles | Median Survival |
| Zhong, Y. | 2014 | 5 | 9.00 | Mouse | Athymic | U87 | Doxorubicin | IV | Multiple | Saline | Nanoparticles | Volume |
| Lin, L. | 2014 | 7 | 16.00 | Mouse | Athymic | U-118MG | Doxorubicin | IV | Multiple | Saline | Nanoliposome | Median Survival |
| Lin, L. | 2014 | 7 | 10.00 | Mouse | Athymic | U-118MG | Doxorubicin | IV | Multiple | Carrier | Nanoliposome | Volume |
| Lin, L. | 2014 | 7 | 10.00 | Mouse | Athymic | U-118MG | Doxorubicin | IV | Multiple | Carrier | Nanoliposome | Volume |
| Kovac, Z. | 2014 | 5 | 5.33 | Mouse | None | GL261 | Doxorubicin | IV | Single | Untreated | Microbubbles | Median Survival |
| Kovac, Z. | 2014 | 5 | 5.67 | Mouse | None | SMA-560 | Doxorubicin | IV | Single | Untreated | Microbubbles | Median Survival |
| Sonabend, A.M. | 2014 | 6 | 21.00 | Mouse | Unknown | Murine glioma | Etoposide | Intracranial | Multiple | Saline | NA | Median survival |
| Sonabend, A.M. | 2014 | 6 | 20.00 | Mouse | Unknown | Murine glioma | Etoposide | Intracranial | Continuous | Saline | NA | Median Survival |
| Sonabend, A.M. | 2014 | 6 | 28.00 | Mouse | Unknown | Murine glioma | Etoposide | Intracranial | Continuous | Saline | NA | Median Survival |
| Tarasenko, N. | 2014 | 6 | 8.40 | Mouse | Athymic | U251 | Doxorubicin | IP | Multiple | Saline | NA | Median Survival |
| Tarasenko, N. | 2014 | 6 | 8.40 | Mouse | Athymic | U251 | Doxorubicin | IP | Multiple | Saline | NA | Volume |
| Jiang, P. | 2014 | 6 | 7.50 | Mouse | Athymic | U87 | Irinotecan | IP | Multiple | Vehicle | NA | Volume |
| Jiang, P. | 2014 | 6 | 7.50 | Mouse | Athymic | U87 | Irinotecan | IP | Multiple | Vehicle | NA | Volume |
| Yang, Y. | 2013 | 5 | 9.33 | Mouse | Athymic | U87 | Doxorubicin | IV | Multiple | Saline | Lipoosme | Median Survival |
| Escoffre, J.M. | 2013 | 5 | 7.50 | Mouse | Athymic | U87 | Irinotecan | IV | Multiple | Untreated | NA | Volume |
| Jaszberenyi, M. | 2013 | 7 | 12.50 | Mouse | Athymic | U87 | Doxorubicin | IV | Continuous | Unknown | NA | Volume |
| Alhenn, D. | 2013 | 6 | 7.50 | Rat | None | F98 | Etoposide |  |  | Carrier | Microspheres | Median Survival |
| Morfouace, M. | 2012 | 3 | 13.33 | Mouse | Athymic | P7CSC | Etoposide | IP | Continuous | Unknown | NA | Volume |
| Munson, J. | 2012 | 4 | 9.00 | Rat | None | eGFP-RT2 | Doxorubicin | IV | Single | Saline | NA | Median Survival |
| Munson, J. | 2012 | 4 | 9.00 | Rat | None | eGFP-RT2 | Doxorubicin | IV | Single | Saline | NA | Volume |
| Cheema, T. | 2011 | 7 | 13.33 | Mouse | Athymic | BT74 | Etoposide | IP | Continuous | Saline | NA | Median Survival |
| Serwer, L. | 2011 | 7 | 9.50 | Mouse | Athymic | U87 | Topotecan | IV | Multiple | Saline | Nanoliposome | Median Survival |
| Serwer, L. | 2011 | 7 | 15.00 | Mouse | Athymic | GBM | Topotecan | IV | Multiple | Saline | Nanoliposome | Median Survival |
| Serwer, L. | 2011 | 7 | 12.00 | Mouse | Athymic | GBM | Topotecan | IV | Multiple | Saline | Nanoliposome | Median Survival |
| Guo, L. | 2011 | 5 | 7.00 | Mouse | Athymic | U87 | Doxorubicin | IV | Multiple | Saline | Lipoosme | Median Survival |
| Lopez, K. | 2011 | 5 | 4.00 | Rat | Unknown | Retrovirus | Topotecan | Intracranial | Single | Saline | NA | Median Survival |
| Lopez, K. | 2011 | 5 | 4.00 | Rat | Unknown | Retrovirus | Topotecan | Intracranial | Multiple | Saline | NA | Median Survival |
| Lopez, K. | 2011 | 5 | 4.00 | Rat | Unknown | Retrovirus | Topotecan | Intracranial | Continuous | Saline | NA | Median Survival |
| Vinchon-Petit, S. | 2010 | 7 | 11.75 | Rat | None | 9L | Doxorubicin | IV | Single | Carrier | Drug-eluting beads | Median survival |
| Panigrahy, D. | 2010 | 6 | 20.00 | Mouse | SCID | U87 | Etoposide | Oral | Continuous | Unknown | NA | Volume |
| Pozsgai, E. | 2000 | 5 | 11.25 | Mouse | Athymic | DBTRG-05 | Doxorubicin | IV | Multiple | Vehicle | NA | Volume |
| Arai, T. | 2010 | 6 | 6.67 | Mouse | Athymic | U87 | Doxorubicin | SubCut | Single | Carrier | Polymer | Volume |
| Kuroda, J. | 2010 | 7 | 5.71 | Mouse | Athymic | U87 | Irinotecan | IV | Multiple | Saline | Micelles | Median Survival |
| Kuroda, J. | 2010 | 7 | 5.71 | Mouse | Athymic | U87 | Irinotecan | IV | Multiple | Saline | Micelles | Median Survival |
| Lu, J. | 2009 | 8 | 6.67 | Mouse | SCID | U87 | Doxorubicin | IP | Multiple | Saline | NA | Volume |
| Hekmatara, T. | 2009 | 8 | 28.00 | Rat | None | 101/8 glioblastoma | Doxorubicin | IV | Multiple | Untreated | Nanoparticles | Volume |
| Kuroda, J. | 2009 | 5 | 9.00 | Mouse | Athymic | U87 | Irinotecan | Intracranial | Multiple | Saline | Micelles | Median Survival |
| Kuroda, J. | 2009 | 5 | 4.50 | Mouse | Athymic | U87 | Irinotecan | SubCut | Multiple | Saline | Micelles | Volume |
| Kreuter, J. | 2008 | 3 | 7.50 | Rat | Unknown | 101/8 | Doxorubicin | IV | Multiple | Untreated | Nanoparticles | Median Survival |
| Petri, B. | 2007 | 7 | 23.33 | Rat | None | 101/8 | Doxorubicin | IV | Multiple | Untreated | Nanoparticles | Median Survival |
| Ambruosi, A. | 2006 | 6 | 11.67 | Rat | None | 101/8 | Doxorubicin | IV | Multiple | Untreated | Nanoparticles | Median Survival |
| Gomez-Manzano, C. | 2006 | 5 | 36.00 | Mouse | Athymic | U87 | Irinotecan | IP | Multiple | Vehicle | Nanoparticles | Median survival |
| Gomez-Manzano, C. | 2006 | 5 | 58.00 | Mouse | Athymic | U87 | Irinotecan | IP | Multiple | Vehicle | Nanoparticles | Median survival |
| Mamot, C. | 2005 | 5 | 12.50 | Mouse | Athymic | U87 | Doxorubicin | IV | Multiple | Saline | Lipoosme | Volume |
| Mamot, C. | 2005 | 5 | 12.50 | Mouse | Athymic | U87 | Epirubicin | IV | Multiple | Saline | Lipoosme | Volume |
| Lesniak, M. | 2005 | 7 | 15.00 | Rat | None | 9L | Doxorubicin | Intracranial | Single | Carrier | Polymer | Median survival |
| Lesniak, M. | 2005 | 7 | 15.00 | Rat | None | 9L | Doxorubicin | Intracranial | Single | Carrier | Polymer | Median survival |
| Steiniger, S. | 2004 | 6 | 27.20 | Rat | None | 101/8 | Doxorubicin | IV | Multiple | Untreated | Nanoparticles | Median Survival |
| Steiniger, S. | 2004 | 6 | 25.60 | Rat | None | 101/8 | Doxorubicin | IV | Multiple | Carrier | Nanoparticles | Median Survival |
| Steiniger, S. | 2004 | 6 | 10.50 | Rat | None | 101/8 | Doxorubicin | IV | Multiple | Untreated | Nanoparticles | Median Survival |
| Prasad, G. | 2002 | 4 | 6.00 | Mouse | Athymic | U87 | Irinotecan | IP | Multiple | Saline | NA | Volume |
| Houghton, P. | 2000 | 4 | 11.63 | Mouse | None | GBM | Irinotecan | IV | Multiple | Untreated | NA | Volume |
| Sharma, U. | 1997 | 5 | 11.00 | Rat | None | 9L | Doxorubicin | IV | Multiple | Saline | Lipoosme | Median Survival |
| Sharma, U. | 1997 | 5 | 9.00 | Rat | None | 9L | Doxorubicin | IV | Multiple | Saline | Lipoosme | Median Survival |
| Sharma, U. | 1997 | 5 | 9.50 | Rat | None | 9L | Doxorubicin | IV | Multiple | Saline | Lipoosme | Median Survival |
| Pechman, K. | 2012 | 4 | 8.60 | Rat | Athymic | U87 | Irinotecan | IV | Single | Untreated | NA | Volume |
| Pechman, K. | 2012 | 4 | 8.60 | Rat | Athymic | U87 | Irinotecan | IV | Single | Untreated | NA | Volume |
| Pechman, K. | 2012 | 4 | 8.60 | Rat | Athymic | U87 | Irinotecan | IV | Single | Untreated | NA | Volume |
| Glage, S. | 2011 | 9 | 16.00 | Rat | None | BT4Ca | Doxorubicin | Intracranial | Single | Carrier | Drug-eluting beads | Median survival |
| Glage, S. | 2011 | 9 | 17.00 | Rat | None | BT4Ca | Doxorubicin | Intracranial | Single | Carrier | Drug-eluting beads | Median survival |
| Glage, S. | 2011 | 9 | 18.00 | Rat | None | BT4Ca | Irinotecan | Intracranial | Single | Carrier | Drug-eluting beads | Median survival |
| Glage, S. | 2011 | 9 | 15.00 | Rat | None | BT4Ca | Doxorubicin | Intracranial | Single | Carrier | Drug-eluting beads | Volume |
| Glage, S. | 2011 | 9 | 13.00 | Rat | None | BT4Ca | Doxorubicin | Intracranial | Single | Carrier | Drug-eluting beads | Volume |
| Glage, S. | 2011 | 9 | 14.00 | Rat | None | BT4Ca | Irinotecan | Intracranial | Single | Carrier | Drug-eluting beads | Volume |
| Verreault, M. | 2012 | 5 | 7.20 | Mouse | RAG2-M | U251 | Doxorubicin | IV | Multiple | Untreated | Lipoosme | Median Survival |
| Verreault, M. | 2012 | 5 | 7.20 | Mouse | RAG2-M | U251 | Doxorubicin | IV | Multiple | Untreated | Lipoosme | Median Survival |
| Baltes, S. | 2010 | 7 | 34.00 | Rat | None | BT4Ca | Irinotecan | Intracranial | Single | Carrier | Drug-eluting beads | Median Survival |
| Baltes, S. | 2010 | 7 | 25.00 | Rat | None | BT4Ca | Doxorubicin | Intracranial | Single | Carrier | Drug-eluting beads | Median Survival |
| Recinos, V.R. | 2010 | 7 | 8.00 | Rat | None | 9L | Epirubicin | IP | Multiple | Untreated | Polymer | Median survival |
| Manome, Y. | 2006 | 5 | 7.50 | Rat | None | RT2 | Doxorubicin | SubCut | Single |  | Polymer | Volume |
| Hsu, W. | 2005 | 7 | 20.33 | Rat | None | 9L | Doxorubicin | Intracranial | Single | Carrier | Polymer | Median survival |
| Morita, K. | 2003 | 3 | 9.60 | Rat | None | C6 | Doxorubicin | IV | Single | Untreated | NA | Median Survival |
| Morita, K. | 2003 | 3 | 9.60 | Rat | None | C6 | Doxorubicin | IArterial | Single | Untreated | NA | Median Survival |
| Chen, P.Y. | 2013 | 7 | 10.80 | Mouse | Athymic | GBM | Irinotecan | IV | Multiple | Untreated | NA | Median Survival |
| Chen, P.Y. | 2013 | 7 | 10.80 | Mouse | Athymic | GBM | Irinotecan | IV | Multiple | Untreated | NA | Median Survival |
| Chen, P.Y. | 2013 | 7 | 10.80 | Mouse | Athymic | GBM | Irinotecan | Intracranial | Single | Untreated | NA | Median Survival |
| Chen, P.Y. | 2013 | 7 | 10.80 | Mouse | Athymic | GBM | Irinotecan | Intracranial | Multiple | Untreated | NA | Median Survival |
| Chen, P.Y. | 2013 | 7 | 8.80 | Mouse | Athymic | GBM | Irinotecan | Intracranial | Multiple | Untreated | NA | Median Survival |
| Hosokawa, Y. | 2015 | 4 | 6.67 | Mouse | Athymic | U251 | Doxorubicin | IP | Continuous | Saline | NA | Volume |
| Li, J. | 2015 | 6 | 16.00 | Mouse | Athymic | U87 | Doxorubicin | IV | Multiple | Saline | Polymer | Median Survival |
| Zhang, C.X. | 2015 | 7 | 7.20 | Mouse | Athymic | U251 | Epirubicin | IV | Multiple | Saline | Lipoosme | Median Survival |
| Verreault, M. | 2015 | 5 | 7.20 | Mouse | SCID | U251 | Doxorubicin | IV | Multiple | Untreated | NA | Median Survival |
| Verreault, M. | 2015 | 5 | 7.50 | Mouse | SCID | U251 | Doxorubicin | IV | Multiple | Untreated | NA | Median Survival |
| Zhao, Y. | 2016 | 7 | 8.00 | Mouse | Athymic | U87 | Doxorubicin | IV | Multiple | Saline | Lipoosme | Median survival |
| Byeon, H.J. | 2016 | 6 | 10.67 | Mouse | Athymic | U87 | Doxorubicin | IV | Multiple | Saline | Nanoparticles | Median Survival |
| Ramachandran, C. | 2016 | 4 | 26.67 | Mouse | Athymic | U87 | Irinotecan | IP | Continuous | Vehicle | NA | Median Survival |
| Ramachandran, C. | 2016 | 4 | 26.67 | Mouse | Athymic | U87 | Irinotecan | IP | Continuous­­ | Vehicle | NA | Volume |

Notice some of the references are duplicated which means that the study included multiple experiments with different study characteristics (i.e. glioma model, type of control, outcome measure, etc.).
