# Supplementary material for: A systematic review and meta-analysis of topoisomerase inhibition in pre-clinical glioma models
Source: Oncotarget. 2018 Jan 29;9(13):11387–401. doi: 10.18632/oncotarget.24334 (PMC5834287; doi:10.18632/oncotarget.24334)
Supplement: Supplementary file 3 [file oncotarget-09-11387-s003.docx]

**Supplementary Table 2: Study quality scores.** Each of the publication was scored according to a 12-item checklist, as listed below, to determine publication bias.

1. Peer-reviewed publication

2. Sample size calculation

3. Randomised allocation of drug (or control) treatment

4. Blinded assessment of outcome

5. Compliance with animal welfare regulations

6. Statement of conflict of interests

7. Uniform number of cells implanted

8. Site of implantation is consistent in all animals

9. “Take rates” of implanted tumor cells is mentioned in the publication

10. Number of excluded animals must be stated with reasons for exclusion mentioned

11. Drug action justified

12. Drug-carrier justified


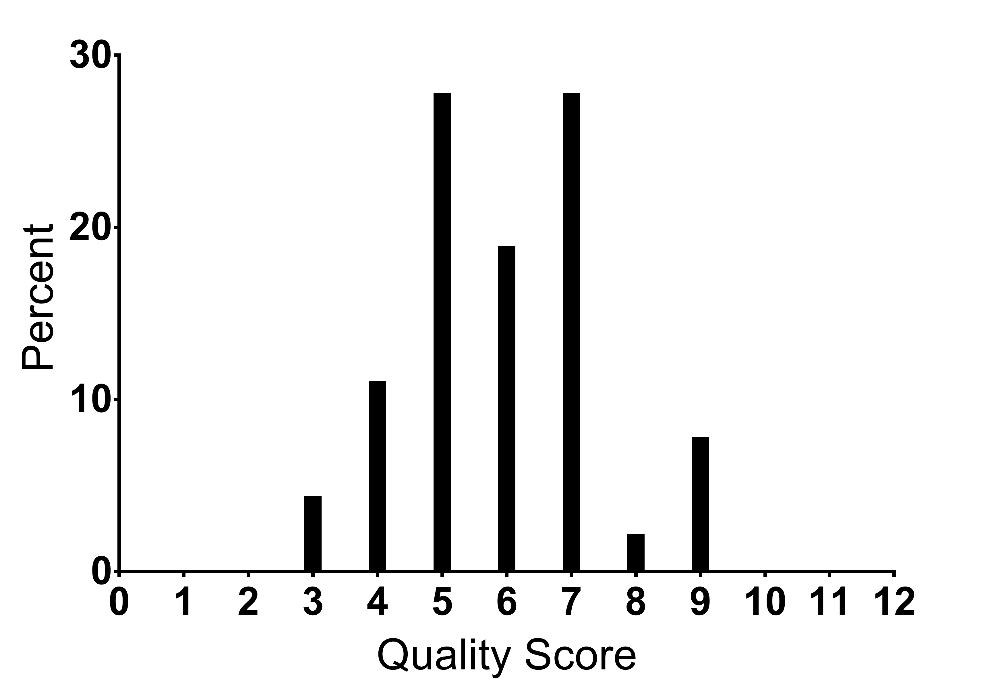


| **Name** | **Year** | **1** | **2** | **3** | **4** | **5** | **6** | **7** | **8** | **9** | **10** | **11** | **12** | **Quality Score** |
| --- | --- | --- | --- | --- | --- | --- | --- | --- | --- | --- | --- | --- | --- | --- |
| Wang, W. | 2015 | + |  | + |  | + | + | + | + |  |  |  |  | 6 |
| Marrero, L. | 2014 | + |  | + |  | + | + | + | + | + |  | + | + | 9 |
| Zhong, Y. | 2014 | + |  |  |  | + |  | + | + |  |  |  | + | 5 |
| Lin, J. | 2014 | + |  | + |  | + | + | + | + |  |  |  | + | 7 |
| Kovac, Z. | 2014 | + |  | + |  | + |  | + | + |  |  |  |  | 5 |
| Sonabend, A. | 2014 | + |  | + |  | + |  |  | + |  |  | + | + | 6 |
| Tarasenko, N. | 2014 | + |  | + |  | + |  | + | + | + |  |  |  | 6 |
| Jiang, P. | 2014 | + |  | + |  | + | + | + | + |  |  |  |  | 6 |
| Yang, Y. | 2013 | + |  |  |  | + |  | + | + |  |  |  | + | 5 |
| Escoffre, J.M. | 2013 |  |  |  |  | + |  | + | + |  |  | + | + | 5 |
| Jaszberenyi, M. | 2013 | + |  | + |  | + | + | + | + | + |  |  |  | 7 |
| Alhenn, D. | 2013 | + |  |  |  | + | + | + | + |  |  | + |  | 6 |
| Morfouace, M. | 2012 | + |  |  |  |  |  | + | + |  |  |  |  | 3 |
| Munson, J. | 2012 | + |  |  |  | + |  | + | + |  |  |  |  | 4 |
| Cheema, T. | 2011 | + |  | + |  | + | + | + | + |  |  | + |  | 7 |
| Serwer, L. | 2011 | + |  | + |  | + | + | + | + |  |  |  | + | 7 |
| Guo, L. | 2011 | + |  |  |  | + |  | + | + |  |  | + |  | 5 |
| Lopez, K. | 2011 | + |  |  |  |  | + |  | + |  |  | + | + | 5 |
| Vinchon-Petit, S. | 2010 | + |  | + |  |  | + | + | + | + |  |  | + | 7 |
| Panigrahy, D. | 2010 | + |  | + |  | + |  | + | + |  |  | + |  | 6 |
| Pozsgai, E. | 2000 | + |  | + |  | + |  |  | + |  | + |  |  | 5 |
| Arai, T. | 2010 | + |  | + |  | + |  | + | + |  |  |  | + | 6 |
| Kuroda, J. | 2010 | + |  | + |  | + | + | + | + |  |  |  | + | 7 |
| Lu, J. | 2009 | + |  | + |  | + | + | + | + | + |  | + |  | 8 |
| Hekmatara, T. | 2009 | + |  | + | + | + |  | + | + | + |  |  | + | 8 |
| Kuroda, J. | 2009 | + |  | + |  | + |  | + |  |  |  |  | + | 5 |
| Kreuter, J. | 2008 | + |  |  |  |  |  |  | + |  |  |  | + | 3 |
| Petri, B. | 2007 | + |  | + |  | + |  | + | + | + |  |  | + | 7 |
| Ambruosi, A. | 2006 | + |  | + |  | + |  | + | + |  |  |  | + | 6 |
| Gomez-Manzano, C. | 2006 | + |  |  |  | + |  | + | + |  |  | + |  | 5 |
| Mamot, C. | 2005 | + |  | + |  |  |  |  | + | + |  |  | + | 5 |
| Lesniak, M. | 2005 | + |  |  |  | + |  | + | + | + |  | + | + | 7 |
| Steiniger, S. | 2004 | + |  | + |  | + |  | + | + | + |  |  |  | 6 |
| Prasad, G. | 2002 | + |  | + |  |  |  | + | + |  |  |  |  | 4 |
| Houghton, P. | 2000 | + |  |  |  | + |  |  | + |  |  | + |  | 4 |
| Sharma, U. | 1997 | + |  |  |  | + |  | + | + |  |  |  | + | 5 |
| Pechman, K. | 2012 | + |  |  |  | + |  | + | + |  |  |  |  | 4 |
| Glage, S. | 2011 | + |  | + | + | + | + | + | + |  | + |  | + | 9 |
| Verreault, M. | 2012 | + |  |  |  | + |  | + | + |  |  |  | + | 5 |
| Baltes, S. | 2010 | + |  | + | + | + |  | + | + |  |  |  | + | 7 |
| Recinos, V.R. | 2010 | + |  | + |  | + |  |  | + | + |  | + | + | 7 |
| Manome, Y. | 2006 | + |  |  |  | + |  | + | + |  |  |  | + | 5 |
| Hsu, W. | 2005 | + |  |  |  | + | + | + | + |  |  | + | + | 7 |
| Morita, K. | 2003 | + |  |  |  |  |  | + | + |  |  |  |  | 3 |
| Chen, P.Y. | 2013 | + |  |  |  | + |  | + | + | + | + |  | + | 7 |
| Hosokawa, Y. | 2015 |  |  | + |  | + |  | + | + |  |  |  |  | 4 |
| Li, J. | 2015 | + |  |  |  | + | + | + | + |  |  |  | + | 6 |
| Zhang, C.X. | 2015 | + |  | + |  | + | + | + | + |  |  |  | + | 7 |
| Verreault, M. | 2015 | + |  |  |  | + |  | + | + | + |  |  |  | 5 |
| Zhao, Y. | 2016 | + |  | + |  | + |  | + | + |  |  | + | + | 7 |
| Byeon, H.J. | 2016 | + |  | + |  | + |  | + | + |  |  |  | + | 6 |
| Ramachandran, C. | 2016 |  |  |  |  | + | + | + | + |  |  |  |  | 4 |
